# Image-Based Morphometric Analysis of Human Milk Fat Globules Versus Laser Diffraction

**DOI:** 10.3390/foods15071205

**Published:** 2026-04-02

**Authors:** Diana Escuder-Vieco, Kristin Keller, Noelia Ureta-Velasco, Clara Alonso-Díaz, María López Cerdán, Carmen Rosa Pallás-Alonso, Nadia Raquel García-Lara

**Affiliations:** 1 Aladina-MGU Regional Human Milk Bank, 12 de Octubre University Hospital, imas12, 28041 Madrid, Spain; biol.kristin.keller@gmail.com (K.K.); nadiaraquelg.nrgl@gmail.com (N.R.G.-L.); 2 Spanish Network in Maternal, Neonatal, Child and Developmental Health Research (RICORS-SAMID, RD24/0013/0008), Instituto de Salud Carlos III, 28029 Madrid, Spain; claraalonsodiaz@gmail.com (C.A.-D.); kpallas.hdoc@gmail.com (C.R.P.-A.); 3 Department of Neonatology, 12 de Octubre University Hospital, 28041 Madrid, Spain; noelia.ureta@gmail.com (N.U.-V.); mlcerdan@salud.madrid.org (M.L.C.)

**Keywords:** breast milk, milk fat globules, agglomerates, morphometric analysis, laser diffraction analysis, Bland–Altman analysis, method comparison, Lin’s concordance correlation coefficient

## Abstract

Human milk fat globules (MFGs)’ size characterization is key for evaluating milk quality and processing effects. Laser diffraction (LD) is widely used for particle size analysis but provides limited morphological information. This study applied image-based morphometric analysis (IBMA) to characterize MFGs’ size and shape distributions in human milk and compared the results with LD measurements. Milk samples from 12 women delivering term and preterm infants were analyzed. LD was performed using a Mastersizer 3000 (Malvern Panalytical, Malvern, UK) and IBMA using a Morphologi 4 (Malvern Panalytical, Malvern, UK), acquiring 2D images at 20× magnification covering particle sizes from ~1.5 to 130 µm. IBMA classified MFGs as individual particles (IP) (HS circularity ≥ 0.920; circle equivalent diameter < 25 µm) or agglomerates (HS circularity < 0.920; solidity < 0.970), extracting descriptors including circularity, elongation, and solidity. IP predominated, while agglomerates represented ~15% of particles. Number-mean diameters (D[1,0]) were 4.91 µm (total), 4.36 µm (IP), and 8.00 µm (agglomerates). Volume-weighted particle diameters (D[4,3]) were 7.21 µm for IP and 14.02 µm for agglomerates. The highest level of agreement between methods was observed for IP D[4,3], although minor differences may be clinically relevant. IBMA and LD provide complementary information; however, IBMA uniquely enables the characterization of MFG structural organization, including the identification of agglomerates, which cannot be resolved by LD. This added level of structural detail may have important implications for understanding the digestibility of human milk, particularly in preterm populations.

## 1. Introduction

Human milk lipids are key nutrients that provide over half of a neonate’s energy needs and play a fundamental role in the development of the nervous and immune systems, lipid metabolism, and the functionality of cellular membranes [1,2]. Lipids are transported within the aqueous phase of milk as milk fat globules (MFGs), which consist of triacylglycerol droplets surrounded by a complex tri-layered membrane [1,3]. The mean size of MFGs is highly heterogeneous, ranging from 0.2 to 15 µm, and this variability is functionally critical [4,5,6].

Smaller MFGs (<3 µm) possess a higher surface-area-to-volume ratio, resulting in a greater proportion of bioactive-rich MFG membrane per unit of fat [2,6,7,8]. This increased surface area not only enhances the bioavailability of bioactive nutrients—such as docosahexaenoic acid, eicosapentaenoic acid, and fat-soluble vitamins—but also provides a more extensive interface for gastric and pancreatic lipase activity [1,2,9]. This is particularly advantageous for preterm infants with immature digestive systems [2,10,11]. Conversely, larger MFGs (e.g., 10–20 µm) are less efficiently digested, which can reduce overall nutrient absorption [2,10]. Consequently, MFG size and surface composition are key determinants of lipid digestion and absorption, directly influencing nutrient bioavailability. Recent evidence has further emphasized the importance of lipid emulsion structure and organization in modulating digestion behavior and physiological responses [12].

From an analytical perspective, accurate characterization of human MFG size distribution is fundamental for evaluating both the nutritional quality and processing-related properties of milk. Recent advances in the microstructural characterization of dairy systems have further emphasized the importance of linking particle size and morphology with functional and processing-related properties [13]. Static light scattering is the most widely used technique for particle size analysis in such systems [5,14,15,16,17,18,19,20]. However, human milk is a highly polydisperse and complex biological matrix, containing MFGs, casein micelles, and protein aggregates. Because the intensity of scattered light scales with the sixth power of particle diameter (R^6^), larger particles dominate the signal, potentially masking smaller particles and biasing the measured size distribution [14,21].

In this context, microscopy-based approaches have gained increasing interest as complementary or alternative techniques. Traditional optical microscopy was previously limited by resolution constraints and labor-intensive manual analysis, but advances in optical systems and the integration of automated high-throughput image analysis have greatly improved its capabilities [22]. Modern computational power and advanced algorithms now allow rapid, automated detection and characterization of thousands of particles within a reasonable timeframe [23]. While transmission and scanning electron microscopy can provide detailed submicron visualization of MFGs, their use is restricted by high operational costs, complex sample preparation, and low analytical throughput [14,21].

Building upon these advances, image-based morphometric analysis (IBMA) enables automated quantification of particle size, shape, and optical properties from digital images. A wide range of descriptors, including particle diameter, circularity, convexity, elongation, solidity, and intensity can be extracted from large particle populations, providing detailed information on MFG morphology and heterogeneity. In this study, IBMA was applied to comprehensively characterize MFGs in human milk, assessing both size and shape distributions. Additionally, IBMA-derived measurements were compared with those obtained by laser diffraction (LD) to evaluate the level of agreement, identify potential sources of divergence, and explore the complementarity of these techniques, providing a more complete understanding of milk fat structure.

## 2. Materials and Methods

### 2.1. Human Milk Samples

Mature human milk samples (5 mL) were collected from 12 women. Three samples were obtained from mothers of preterm infants hospitalized in the Neonatology Unit of Hospital 12 de Octubre, while nine samples were collected from mothers of full-term infants who were donors to the Regional Human Milk Bank of the Community of Madrid. Each sample corresponded to a complete expression from a single breast. The samples were stored frozen (−25 °C) until analysis. The study was conducted in accordance with the principles outlined in the Declaration of Helsinki (1975, revised in 2013), and approved by the Clinical Research Ethics Committee of “Hospital 12 de Octubre” (protocol code 21/202). Written informed consent was obtained from all participants.

### 2.2. Particle Size Distribution by LD

MFG size distribution was determined by LD using a Mastersizer 3000(Malvern Panalytical, Malvern, UK), equipped with a He–Ne laser operating at 633 nm and an electroluminescent diode at 466 nm and analyzed using Mastersizer Xplorer software (v5.02) (Malvern Panalytical, Malvern, UK). The measurable particle size range was approximately 0.01–3500 µm. Prior to analysis, frozen samples were thawed and diluted (1:1, *v*/*v*) with 35 nmol L^−1^ EDTA/NaOH buffer (pH 7.0) (Sigma-Aldrich, St. Louis, MO, USA) to dissociate casein micelles and protein aggregates (Michalski, 2005) [5]. Samples were introduced into the dispersion unit (Hydro EV, Malvern Panalytical, Malvern, UK) which was filled with distilled water using a precision pipette until an obscuration level of 8–10% was achieved. The stirrer speed was set to 1700 rpm. Refractive indices were set at 1.46 for milk fat and 1.33 for water, according to literature values [5]. Measurements were performed in triplicate for each sample to ensure reproducibility. Particle size distributions were calculated using Mie theory and expressed on a volume basis. Individual particle size was characterized by the volume-equivalent spherical diameter.

### 2.3. Particle Size Distribution and Morphological Characterization by IBMA

#### 2.3.1. Human Milk Sample Preparation

Breast milk samples were thawed at 37 °C and, when fat content exceeded 3.5 g dL^−1^, diluted 1:4 (*v*/*v*) in Milli-Q water, obtained using a Milli-Q purification system (Merck Millipore, Darmstadt, Germany), to minimize particle overlap and optimize image clarity. Fat content was determined by Fourier-transform mid-infrared spectroscopy (FOSS, Hillerød, Denmark). A thin film was prepared by depositing 20 µL of human milk onto a clean glass slide Thermo Fisher Scientific (Waltham, MA, USA) and spreading it with a second slide, following a procedure similar to that used for peripheral blood smears. Samples were air-dried for a few seconds at room temperature and subsequently placed under the IBMA device for image acquisition.

#### 2.3.2. Imaging Method

MFG particle size and morphology were analysed using the Morphologi 4 (Malvern Panalytical, Malvern, UK), an automated optical imaging system equipped with a Nikon CFI60 and a high-resolution 18 MP colour camera (4912 × 3684 pixels, 1.25 µm pixel size). Samples were illuminated using a white LED light source with configurable bright field and dark field modes.

Images were acquired by a 20× magnification objective, covering particle size ranges from approximately 1.5 to 130 μm. Particle detection and segmentation were performed automatically using the integrated Sharp Edge algorithm. At least 70,000 particles were analysed per sample to ensure statistical robustness. The system was operated under standard operating procedures (SOPs) to ensure consistent lighting, focus, and thresholding conditions across all samples. The SOPs was adjusted to the following parameters: (1) Magnification = 20 × (1.5 − 130 μm), (2) Minimum number of pixels = 25, (3) Calibration intensity = 80%, (4) Intensity tolerance = 0.20 (5) Scan area = 25 mm^2^, and (6) Maximum numbers of particles = 200,000. One measurement was performed for each sample.

#### 2.3.3. Image Analysis

Static two-dimensional images were processed using Morphologi 4 software (Version 10.32, Malvern Panalytical, Malvern, UK) to obtain particle size and morphological descriptors. IBMA provided number-based particle size distributions and circle equivalent (CE) diameter metrics. In addition to size parameters, multiple morphological descriptors were extracted to further characterize particle area and shape.

The evaluated parameters included particle area (pixels), intensity standard deviation (intensity SD), high-sensitivity circularity (HS circularity), convexity, elongation, and solidity. HS circularity was calculated as 4π × area/perimeter^2^. Convexity was defined as the ratio between the convex hull perimeter and the actual particle perimeter. Elongation was calculated as 1 − (width/length), and solidity as the ratio between particle area and convex hull area. HS circularity, convexity, and solidity values ranged from 0 to 1, where values closer to 1 indicate a more circular shape, higher convexity, and greater filling of the convex hull, respectively. Elongation values approaching 1 indicate highly elongated particles. Intensity SD describes the variation in grayscale values within each particle (scale 0–255), where lower values indicate more uniform internal intensity and higher values indicate greater internal contrast [21,24].

### 2.4. Statistical Analysis

Results from both analytical methods are presented as mean ± standard deviation (SD). The IBMA technique provided a direct measurement of the number-weighted particle size distribution, from which CE diameter metrics were obtained (D[1,0], Pn10, Pn50, and Pn90). Volume-weighted metrics (D[4,3], Pv10, Pv50, and Pv90) were subsequently derived using the instrument software to enable direct comparison with LD measurements, which are inherently volume-weighted. In addition, the direct assessment of morphological descriptors by IBMA allowed particles to be grouped into three categories: total particles, individual particles, and agglomerates. Differences in CE diameter metrics and morphology parameters between individual particles and agglomerates were assessed by paired Student’s *t*-test.

We further evaluated the influence of baseline characteristics on the relative proportion (%) of agglomerates and on particle size distribution metrics obtained by IBMA and LD. Statistical significance was determined using Pearson correlation coefficients for continuous variables and two-sample Student’s *t*-tests for dichotomous variables.

Multiple approaches were applied to compare volume-weighted particle size metrics (D[4,3], Pv10, Pv50, and Pv90) obtained by LD with those derived from the three IBMA particle classifications (total particles, individual particles, and agglomerates). Linear association between methods was evaluated using the Pearson correlation coefficient (r). Systematic bias was assessed using paired Student’s *t*-tests to determine whether the mean inter-method difference significantly deviated from zero.

The level of agreement and accuracy were further evaluated using Lin’s concordance correlation coefficient (CCC; r_c_), which quantifies deviation from the line of identity. Concordance plots were constructed by plotting LD measurements against IBMA measurements, including both the line of best fit and the identity line (y = x).

Inter-method agreement was additionally assessed using Bland–Altman analysis, in which the difference between paired measurements was plotted against their mean. Mean bias and 95% limits of agreement were calculated to characterize inter-method variability.

Proportional bias was examined using linear regression analysis with the inter-method difference as the dependent variable and the paired mean as the independent variable. A regression slope significantly different from zero (*p* < 0.05) was interpreted as evidence of proportional bias.

All statistical analyses were performed using SPSS (IBM SPSS Statistics, versions 19 and 26; IBM Corp., Chicago, IL, USA). Data distribution was evaluated using the Shapiro–Wilk test to guide test selection. To improve the robustness of the statistical analysis given the small sample size (*n* = 12), significance for Pearson correlations and *t*-tests was assessed using 3000 bias-corrected and accelerated (BCa) bootstrap resamples. Bootstrapping is a non-parametric resampling technique in which the original dataset is treated as a population, and multiple samples are repeatedly drawn with replacement to generate empirical distributions of the estimates. The BCa method was used to derive 95% confidence intervals, as it accounts for both bias and skewness in the data, which are common in biological particle size distributions. Statistical significance was inferred when the 95% confidence interval did not include zero. This approach improves the stability and reliability of the estimates under small-sample conditions without relying on large-sample assumptions. Statistical significance was set at *p* < 0.05, and unadjusted *p*-values are reported. No formal correction for multiple hypothesis testing was applied, as the analyses were exploratory in nature and aimed at identifying patterns of agreement and differences between methods rather than drawing confirmatory conclusions. It is also important to note that, while bootstrapping enhances the robustness of the estimates, it does not increase statistical power nor account for potential biases inherent to the sample. Consequently, the limited sample size remains an important constraint of the study.

## 3. Results

### 3.1. Characteristics of Mothers, Infants, and Human Milk Samples

The baseline characteristics of the women, their infants, and the human milk sampling process are presented in Table 1. The participating women exhibited a wide range of breastfeeding durations, from 0.3 to 18.8 months, and ages ranging from 19 to 41 years. Among their infants, 58.3% (*n* = 7) were female. Human milk samples (*n* = 12) were collected and, in some cases, diluted prior to IBMA (four samples, 33.3%). The milk was stored at −25 °C until analysis, with an average storage time of 14.7 ± 7.3 days, and no sample was stored for longer than one month.

### 3.2. Morphological Characterization and Number-Based Particle Size Distribution by IBMA

Images captured using the IBMA system were first visually inspected, after which quantitative filters were applied to exclude foreign particles or particles of low image quality. Particles with convexity < 0.750, solidity < 0.700, area < 100 pixels, or intensity SD < 10 were excluded.

**Figure 1:**
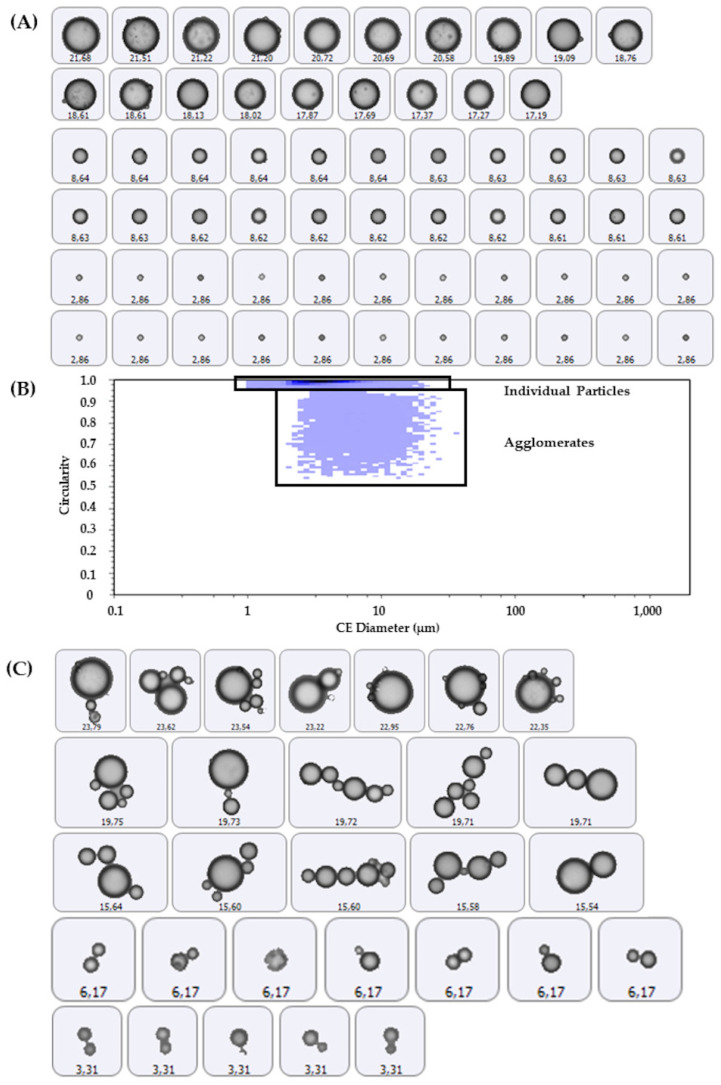
Example from Woman 2 showing human milk fat globule size distribution and particle classification using image-based morphometric analysis. (**A**) Representative 2D grayscale images of fat globules with varying D[1,0], classified as individual particles based on HS circularity ≥ 0.920 and D[1,0] < 25 µm. (**B**) Scatter plot of D[1,0] versus circularity showing the classification regions for individual particles and agglomerates. The blue/purple color gradient represents particle density, where darker shades indicate a higher frequency of fat globules. (**C**) Representative 2D grayscale images of fat globules classified as agglomerates based on HS circularity < 0.920 and solidity < 0.970. The labels on each image indicate the D[1,0] value of each fat globule. Abbreviations: D[1,0], number-based circle equivalent mean diameter; CE, circle equivalent; HS, high-sensitivity.

In addition to filtering, particle classification was performed using the number-weighted CE mean diameter (D[1,0]) and morphological parameters (Figure 1). This classification was based on a protocol developed by Malvern Panalytical, Malvern, UK [22], which combines particle size and shape descriptors (e.g., circularity and solidity) for the identification of agglomerates, and was further adapted to MFG characteristics through expert consultation and visual validation of particle images. Particles were categorized as individual particles or agglomerates based on the following morphological criteria: (1) Individual particles—HS Circularity ≥ 0.920 and CE Diameter < 25 µm; (2) Agglomerates—HS Circularity < 0.920 and Solidity < 0.970; and (3) Total particles—no additional criteria were applied beyond the initial filtering for foreign or low-quality particles. The selected thresholds were chosen to best discriminate between isolated globules and aggregated structures within the specific size range of human milk fat globules.

**Figure 2:**
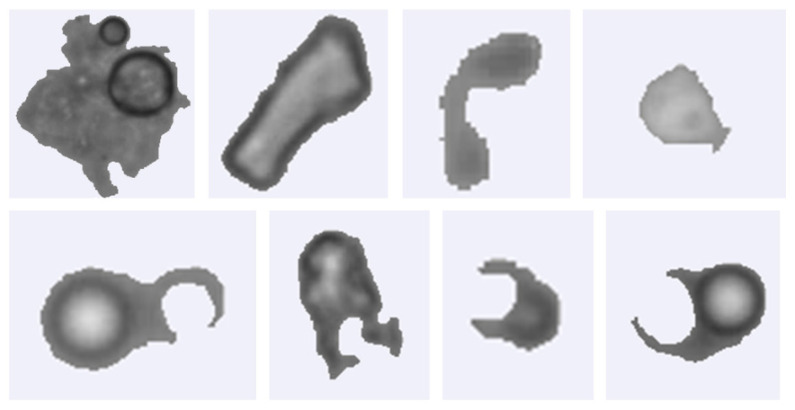
Examples of particles excluded from image-based morphometric analysis due to irregular morphological characteristics.

Particles with unusual morphologies, such as bubbles, membrane remnants, or other irregular shapes, were manually excluded. Examples of these atypical particles are shown in Figure 2. Table 2 summarizes the number and percentage of total particles, individual particles, and agglomerates for each participant. Sample 9 exhibited the highest particle count, with nearly 170,000 particles analyzed, whereas the lowest count was approximately 70,000. Individual particles were the predominant form across all samples. Agglomerates were less abundant, averaging 15% of measured particles and reaching a maximum of 32.1% in sample 12.

Table 3 presents the CE diameter metrics and corresponding morphological parameters of MFGs for all 12 human milk samples, classified by IBMA as total particles, individual particles, or agglomerates. Agglomerates consistently exhibited larger CE mean diameters across all measured metrics compared to individual particles. Morphologically, agglomerates showed lower mean values for HS circularity, solidity, and convexity. Despite this, the overall mean convexity of 0.917 indicates that agglomerates generally retained a substantially convex shape. However, their HS circularity (0.631) was notably lower than 1, reflecting deviation from a perfectly circular form. Agglomerates also displayed higher elongation values than individual particles, and their average intensity SD was slightly higher.

### 3.3. Relationship Between Particle Size Measurement and Baseline Characteristics

To evaluate the influence of baseline characteristics on particle size distribution, we examined the relationships between maternal age, infant sex, breastfeeding duration, birth status (term vs. preterm), storage duration, and sample dilution with the relative proportion of agglomerates and various particle size metrics measured by IBMA (volume- or number-based) and LD.

No significant associations were observed between the percentage of agglomerates and any baseline characteristic. Similarly, particle size metrics from both IBMA and LD showed no significant associations or differences with infant sex, maternal age, breastfeeding duration, or sample dilution.

However, storage duration was significantly associated with several particle size metrics (Table 4). D[1,0], Pn10, and Pn50 showed moderate negative correlations with storage time, whereas D[4,3] in the LD fraction showed a positive association. Additionally, significant differences according to birth status were observed across multiple particle size parameters (Table 4). Overall, full-term samples consistently exhibited larger particle sizes than preterm samples across the different fractions analyzed (individual particles, agglomerates, and LD).

### 3.4. Comparison of Volume-Based Particle Size Metrics Between LD and IBMA

**Figure 3:**
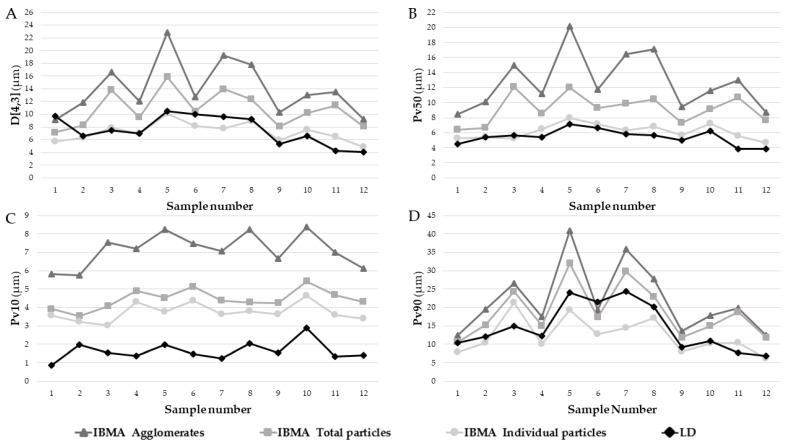
Volume-based milk fat globule particle size diameters of the 12 human milk samples measured by LD and IBMA. For the IBMA data, the particle size diameters were plotted for agglomerates, total particles, and individual particles. The data is presented for four statistical metrics: (**A**) D[4,3], (**B**) Pv50, (**C**) Pv10, and (**D**) Pv90. D[4,3]—volume-based equivalent mean diameter; Pv10, 50, 90: 10th, 50th, and 90th percentiles of the equivalent volume-based particle size distribution. IBMA—image-based morphometric analysis, LD—laser diffraction.

Figure 3 presents a graphical comparison of the volume-based MFG particle size metrics for the 12 human milk samples. Each sample is represented by four data points corresponding to the LD measurement and the three IBMA classifications: individual particles, total particles, and agglomerates. Four statistical measures are shown: D[4,3], Pv10, Pv50, and Pv90.

Overall, the graphs reveal consistent trends across all measures. LD and IBMA individual particle measurements generally yielded lower equivalent particle diameters compared to total particles and agglomerates, indicating that these methods capture smaller particle populations. Agglomerates consistently exhibited the largest particle diameters, while total particle measurements fell between individual particles and agglomerates, with this pattern most clearly observed in Pv10 (Figure 3C). Fluctuations were notable for IBMA total particle and agglomerate measurements in D[4,3] (Figure 3A) and Pv50 (Figure 3B), whereas LD and IBMA individual particles remained more consistent. Pv10 values showed high consistency across methods, although LD values were consistently lower than those for IBMA individual particles (Figure 3C). In contrast, Pv90 exhibited the greatest variability across all methods and classifications (Figure 3D).

**Figure 4:**
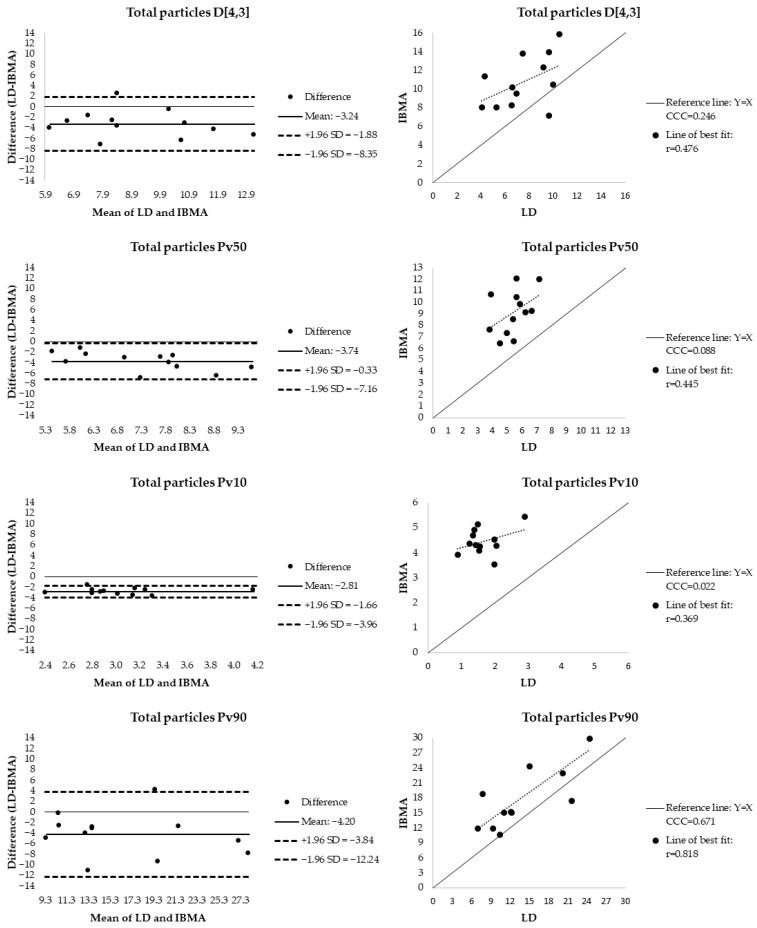
Assessment of agreement and accuracy between LD and IBMA total particles for volume-based particle diameter metrics (D[4,3], Pv50, Pv10, and Pv90) in human milk fat globules. (**Left**): Bland–Altman plots representing the difference (LD—IBMA) against the mean of the two methods, including the mean bias (solid line) and 95% limits of agreement (dashed lines). (**Right**): Concordance correlation plots of LD measurements versus IBMA relative to the line of identity (y = x) (solid line) and the line of best fit (dotted line). D[4,3]—volume-based equivalent mean diameter; Pv10, 50, 90: 10th, 50th, and 90th percentiles of the equivalent volume-based particle size distribution. CCC—Lin’s concordance correlation coefficient, IBMA—image-based morphometric analysis, LD—laser diffraction, SD—standard deviation.

**Figure 5:**
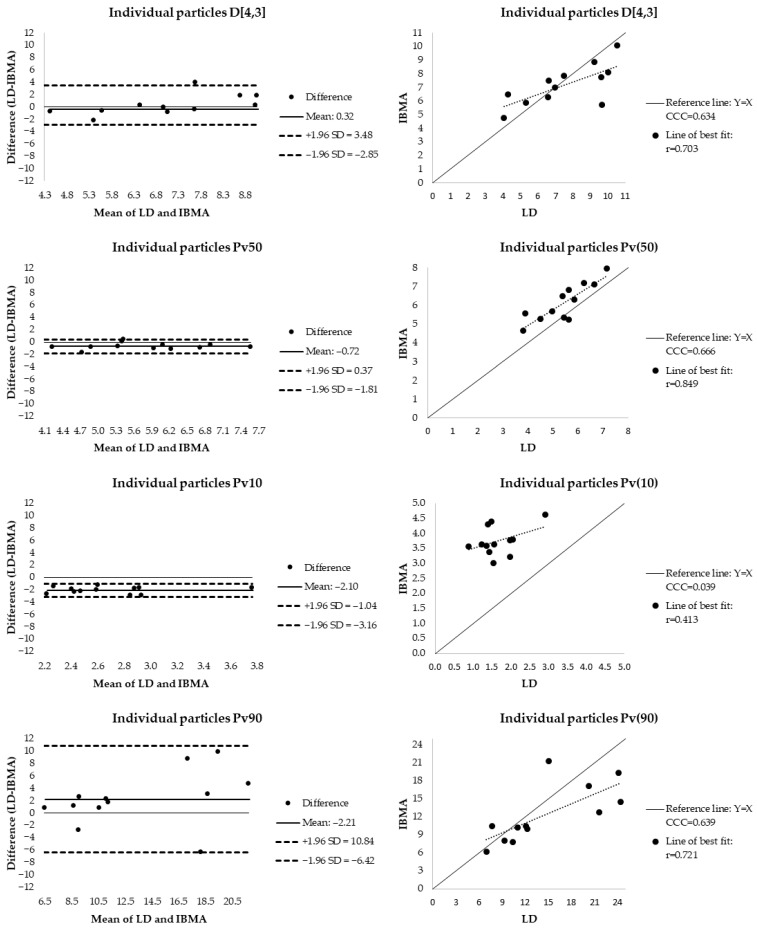
Assessment of agreement and accuracy between LD and IBMA individual particles for volume-based particle diameter metrics (D[4,3], Pv50, Pv10, and Pv90) in human milk fat globules. (**Left**): Bland–Altman plots representing the difference (LD—IBMA) against the mean of the two methods, including the mean bias (solid line) and 95% limits of agreement (dashed lines). (**Right**): Concordance correlation plots of LD measurements versus IBMA relative to the line of identity (y = x) (solid line) and the line of best fit (dotted line). D[4,3]—volume-based equivalent mean diameter; Pv10, 50, 90: 10th, 50th, and 90th percentiles of the equivalent volume-based particle size distribution. CCC—Lin’s concordance correlation coefficient, IBMA—image-based morphometric analysis, LD—laser diffraction, SD—standard deviation.

**Figure 6:**
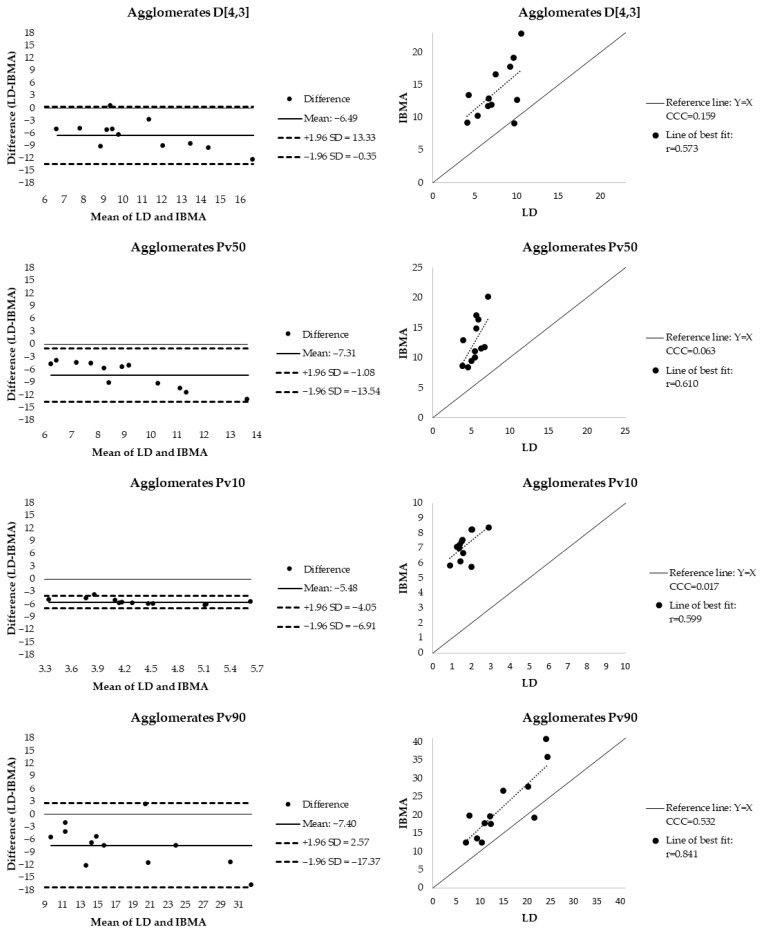
Assessment of agreement and accuracy between LD and IBMA agglomerates for volume-based particle diameter metrics (D[4,3], Pv50, Pv10, and Pv90) in human milk fat globules. (**Left**): Bland–Altman plots representing the difference (LD—IBMA) against the mean of the two methods, including the mean bias (solid line) and 95% limits of agreement (dashed lines). (**Right**): Concordance correlation plots of LD measurements versus IBMA relative to the line of identity (y = x) (solid line) and the line of best fit (dotted line). D[4,3]—volume-based equivalent mean diameter; Pv10, 50, 90: 10th, 50th, and 90th percentiles of the equivalent volume-based particle size distribution. CCC—Lin’s concordance correlation coefficient, IBMA—image-based morphometric analysis, LD—laser diffraction, SD—standard deviation.

Table 5 presents the mean (±SD) of the volume-weighted particle diameter metrics (D[4,3], Pv10, Pv50, and Pv90) assessed by both IBMA and LD. IBMA results are shown for the three particle classifications: total particles, agglomerates, and individual particles. To evaluate the level of agreement between methods, a multi-step statistical approach was applied, including Pearson correlation for linear relationships (Table 5; Figure 4, Figure 5 and Figure 6), paired sample *t*-tests for systematic bias (Table 5), CCC for accuracy (Table 5; Figure 4, Figure 5 and Figure 6), and Bland–Altman plots for limits-of-agreement analysis (Figure 4, Figure 5 and Figure 6). Proportional bias was further assessed via linear regression between the differences and the means of the two methods (Table 5). Additionally, box plots illustrating the comparison between techniques are provided as Supplementary Figures S1–S3.

The highest level of agreement between LD and IBMA for individual particles was observed for the D[4,3] metric. This comparison showed a significant positive correlation (r = 0.703, *p* = 0.011) and no evidence of systematic bias (*p* = 0.505). The CCC (r_c_ = 0.634) indicates moderate agreement relative to the measurement scale. Bland–Altman analysis revealed a small mean difference of 0.32 µm, with 95% limits of agreement ranging from −2.85 to 3.48 µm. Linear regression of the differences confirmed the presence of proportional bias (95% CI: 0.159–1.067).

In contrast, most other metric comparisons showed limited agreement between methods. Significant correlations were observed for some parameters, including Pv90 for total particles (r = 0.818, *p* = 0.001) and Pv90 for agglomerates (r = 0.841, *p* = 0.001). However, despite these linear associations, significant mean differences between methods were detected for nearly all metrics except for D[4,3] and Pv90 in the individual particle class. As shown in Figure 4, Figure 5 and Figure 6, the mean differences were predominantly negative, indicating that IBMA systematically reported larger particle diameters than LD. This discrepancy was most pronounced in the agglomerate class, where the D[4,3] mean difference reached −6.49 µm.

Accuracy assessed by CCC reached moderate levels (>0.6) for D[4,3] (individual), Pv50 (individual), and Pv90 (total and individual). Nevertheless, Bland–Altman analysis revealed wide limits of agreement for Pv90 in all particle classes, indicating considerable inter-method variability and limited practical agreement.

Significant proportional bias was identified for Pv10 (agglomerates), D[4,3] (individual and agglomerates), and Pv50 (total and agglomerates), where the difference between methods systematically changes as the average measurement size increases.

## 4. Discussion

The size distribution of MFGs plays a key role in nutrient bioavailability and metabolic response, particularly in preterm infants, whose digestive and metabolic systems are more immature [2,10,11]. Accurate MFG size characterization is therefore essential for assessing milk quality and processing effects. While LD is the most used method for particle size analysis, this study evaluates IBMA as an alternative approach. Compared to LD, IBMA allows direct visualization of particles, facilitating the identification and distinction of individual globules and agglomerates. Our results show moderate-to-good levels of agreement between IBMA and LD for the mean size of individual particles, while IBMA additionally provides structural information on larger and aggregated particles. These findings support IBMA as a complementary—and in certain applications, alternative—technique for assessing MFGs size distribution in human milk.

Beyond size estimation, IBMA proved particularly useful for assessing the native structure of human MFGs, as samples were analyzed with minimal preparation, reducing structural disruption and allowing globules to be observed in a near-physiological state, including the presence of clusters. In our dataset, agglomerates accounted for an average of 15% of detected particles (range: 8.52–32.07%). These structures were identified through automated morphological classification using a validated agglomerate-identification protocol adapted to human milk and confirmed by visual inspection [24].

Unlike LD, which provides single bulk size outputs, IBMA offers additional structural resolution by distinguishing between different morphological subpopulations, including total particles, individual globules, and agglomerates, allowing a more detailed structural description. Morphological descriptors confirmed that agglomerates differ systematically from individual globules, showing lower circularity and solidity and higher elongation and intensity variability, consistent with irregular but compact clustered structures rather than classification artefacts. This distinction is functionally relevant, since cluster architecture and surface complexity are expected to influence gastric disintegration and lipid digestion, supporting a shift from purely size-based metrics toward broader structural characterization when evaluating milk fat bio-accessibility [25,26].

Although direct evidence for the behavior of these aggregates in vivo is currently limited, in vitro studies suggest that MFG clusters can slow lipase access, modulating the kinetics of lipid hydrolysis and potentially influencing nutrient bioavailability [5,23,24]. These observations provide a mechanistic rationale for a potential functional role of MFG aggregates in digestive efficiency, particularly in preterm infants. Given that cluster architecture and surface complexity may alter enzyme accessibility, further studies—ideally combining in vitro digestion models with in vivo validation—are warranted to clarify their impact on lipid bioavailability and nutrient absorption.

For the overall particle population, the number-based CE mean diameter (D[1,0]) was 4.91 µm, with smaller values for individual globules (4.36 µm) and larger values for agglomerates (8.00 µm). As expected, volume-weighted particle diameters (D[4,3]) were higher (10.76 µm, 7.21 µm, and 14.02 µm, respectively), reflecting the strong influence of large particles in volume-based calculations. A number-based size distribution gives the same statistical weight to every detected particle, regardless of its CE diameter. As a result, the number-based distribution is strongly influenced by the abundant population of small globules, even when their contribution to total fat volume is limited. In contrast, volume-based distribution weights each particle according to its volume, meaning that larger globules and clusters contribute disproportionately to the final metric. Because particle volume scales with the cube of the diameter, even a small fraction of large structures can markedly influence volume-weighted measures. This effect is particularly relevant in heterogeneous systems such as human milk, where individual globules and aggregates coexist. While most published data rely on volume-based metrics derived from LD, number-based descriptors obtained by optical methods such as IBMA or confocal microscopy provide complementary information that may be more closely linked to digestive behavior and lipid bioavailability [2,14,15,16,18,19,20].

Given that MFG size and structure are commonly used to assess the effects of processing and biological factors on milk fat properties [15,16,19,20], IBMA adds value by enabling particle classification and providing both number- and volume-based metrics. This facilitates a more detailed assessment of particle size behavior in relation to sample characteristics. In our study, storage time was negatively associated with IBMA number-based parameters (D[1,0] for total particles, Pn10 for agglomerates and Pn50 for both individual particles and agglomerates), indicating a progressive reduction in the count of detectable large entities, particularly agglomerated structures. In contrast, LD-derived D[4,3] correlated positively with storage duration, suggesting that although total particle counts decrease, the volume-weighted size distribution shifts toward larger particle diameters. This pattern was not observed in IBMA volume-based metrics and likely reflects methodological differences between techniques, highlighting the influence of a limited number of high-volume events on LD-derived estimates. A similar rightward shift in particle size distribution during frozen storage at −18 °C has been previously reported, supporting the plausibility of storage-related enlargement effects [27].

From a physicochemical perspective, these findings are consistent with storage-induced reorganization of the human milk colloidal system. Human milk is a complex oil-in-water emulsion in which globule stability depends on the integrity of the milk fat globule membrane and associated interfacial proteins; structural perturbations during cold storage may facilitate droplet interaction and cluster formation [8,28]. Freezing-related stresses, including ice crystal formation and phase separation, promote cryoconcentration of solutes in the unfrozen phase, thereby increasing particle interactions and favoring aggregation phenomena such as flocculation and partial coalescence in milk systems [5,23]. In addition, structural alterations of the milk fat globule membrane may reduce interfacial stability, further facilitating droplet–droplet interactions and the formation of larger clusters. These processes can reduce the number of discrete particles while increasing the prevalence of aggregated structures, which in turn skews volume-weighted measurements toward larger particles. In this context, the ability of IBMA to distinguish between single enlarged globules and multi-globule agglomerates provides complementary structural information that helps explain the partial discrepancies observed between IBMA and LD. Such behavior is consistent with previous reports on lipid emulsions and dairy systems subjected to freezing and storage stresses [12,13].

The observed differences in particle size distribution between preterm and full-term milk may be related to gestational influences on milk lipid secretion and globule structure. In our study, full-term samples consistently exhibited higher mean particle diameters (D[4,3]), Pv50 and Pv90), aligning with longitudinal maturation trends documented by Jiang et al. (2020) and Zou et al. (2012) [16,18]. Since the preterm mothers in our cohort were in early-lactation stages, their milk structurally was similar to the transitional phase, which is characterized by smaller, more numerous globules [18]. These compositional differences, including higher protein concentrations and distinct bioactive membrane components in preterm milk [2,29,30], significantly influence interfacial properties and emulsion stability. Zou et al. (2012) highlighted that the MFG membrane evolves during the first month [18], affecting the resilience of the lipid structure [8]. Furthermore, variations in the casein-to-whey ratio and mineral content impact colloidal interactions and bridging phenomena between droplets [28]. As Jiang et al. (2020) observed, the evolution of fatty acid profiles during the first 30 days further dictates lipid organization [16]. Ultimately, the smaller MFG size observed in preterm milk is likely influenced by multiple factors beyond digestive adaptation. Immature mammary development and incomplete maturation of lipid synthesis and secretion pathways may limit the formation of large globules, while compositional differences such as higher protein content and distinct membrane-associated bioactive components favor smaller, more numerous MFGs [2,14,16,27]. Functionally, these smaller globules provide a higher surface-to-volume ratio, potentially enhancing lipid accessibility for the underdeveloped digestive systems of preterm infants.

Despite the consistency of these patterns, these findings should be considered preliminary and interpreted with caution given the limited sample size and cohort heterogeneity. Although bootstrapping was applied to improve the robustness of the estimates, confirmation in larger and more balanced populations is still required.

A further objective of this study was to compare IBMA and LD measurements and to assess the level of methodological agreement between the two techniques. For a valid comparison, metrics must be evaluated on the same basis. Although both methods assess the same particle population, they rely on different measurement principles and therefore cannot be expected to yield directly interchangeable results. Because IBMA records individual particle diameters, conversion from number-based to volume-based distributions is methodologically sound and widely accepted [31], and the large number of detected particles in our study (>70,000 across samples) supports robust transformation. However, while both IBMA and LD report volume-based metrics, their underlying geo-metric assumptions differ substantially. IBMA relies on circular equivalent (CE) diameters derived from 2D images for volume estimation, whereas LD calculates sphere-equivalent diameters based on light-scattering patterns in three dimensions. Consequently, although both metrics are expressed as volume-based, they originate from distinct physical principles—2D projections versus 3D light-scattering models. These differences should be considered when comparing results.

LD remains the most widely used technique for MFG size analysis, but it should not be considered the “gold standard” method for human milk. Its bulk optical measurement requires strong dilution and dispersing agents, which disrupt native clusters and primarily reflect individual globules, as described in established protocols [5]. In addition, laser diffraction methods are not fully validated for human milk and commonly apply refractive index parameters derived from bovine fat globules [14]. IBMA, in contrast, preserves cluster structures and provides particle-level morphological information, although it requires careful sample preparation to avoid overlap in high-fat samples.

A visual comparison of trend lines for volume-based equivalent diameter metrics (D[4,3], Pv10, Pv50, and Pv90) across the 12 human milk samples highlights systematic methodological differences between IBMA and LD. Divergence was most evident in the lower percentile range (Pv10), where IBMA showed a higher effective detection limit than LD. Laser diffraction can detect submicron particles (<1 µm) through wide-angle scattering signals, whereas IBMA is constrained by the optical resolution of the imaging system; objects represented by only a few pixels are excluded by segmentation and quality filters. In this study, we selected the use of 20× magnification (measurement range 1.5–130 µm) instead of 50 × (0.5–50 µm), which further shifted sensitivity toward larger particles and cluster detection, at the expense of the smallest size fraction. IBMA therefore better captured upper-percentile sizes (Pv90) and cluster structures that are disrupted during LD preparation. When IBMA individual-particle data were compared with LD outputs, a higher level of agreement was observed for central volume-based metrics such as D[4,3] and Pv50, suggesting closer correspondence between the two techniques when assessing a similarly disaggregated population.

Following the recommendations of Looney (2018) [32], we employed a multi-faceted statistical approach to investigate the level of agreement, including (1) graphical display (Bland–Altman Plot); (2) unscaled indices (95% limits of agreement), and (3) scaled indices (CCC). Furthermore, we provided statistical tests to determine linear relationship, and systematic and proportional bias. The highest level of statistical agreement occurred between LD and IBMA individual particles for the D[4,3] metric. Correlation was significant but moderate, with no evidence of systematic bias, a concordance correlation coefficient of 0.634 [33], and a small mean difference (0.32 µm). However, limits of agreement were relatively wide (ranging from −2.85 to 3.48 µm) and regression analysis indicated proportional bias (95% confidence interval: 0.159, 1.067), with discrepancies increasing at larger particle diameters. These findings suggest statistical association but limited clinical interchangeability. This limitation is relevant because small particle diameter differences translate into substantial surface area differences, directly affecting estimates of lipase accessibility and lipid digestion efficiency [34]. Therefore, IBMA and LD should be considered complementary rather than interchangeable tools for human MFG characterization.

It should be noted that IBMA outputs may be influenced by imaging parameters and particle detection thresholds, which may introduce analytical bias. In this study, imaging was performed at 20× magnification, corresponding to an effective size range of approximately 1.5–130 µm, which inherently limits the detection of submicron particles below the optical resolution and segmentation criteria. Particle detection and segmentation were based on predefined thresholds for intensity, convexity, solidity, and minimum pixel area. While these settings are necessary to exclude artefacts and low-quality detections, they may also lead to the exclusion of small or morphologically atypical particles and influence the lower end of the size distribution. In addition, morphological criteria used for particle classification into individual globules and agglomerates may affect category assignment, particularly near threshold boundaries. Although these parameters were kept constant across all samples to ensure consistency, they may impact absolute size estimates and classification outcomes. These limitations should be considered when interpreting IBMA-derived metrics and when comparing them with laser diffraction, given the differences in measurement principles, resolution, and data processing approaches between techniques.

Further limitations of this study include the small sample size and the heterogeneity of the maternal cohort, which may limit the generalizability of the findings. In addition, no formal correction for multiple hypothesis testing was applied, which may increase the risk of Type I errors; therefore, the results should be interpreted with appropriate caution. Despite these limitations, consistent trends in particle size and morphology were observed across diverse samples, supporting the robustness of the identified patterns. Methodological constraints should also be considered when interpreting the results. The recent adoption of IBMA means that validated reference ranges for specific metrics (e.g., circularity) are still lacking, which restricts comparability with existing literature. In addition, the lack of a standardized human milk preparation protocol for IBMA introduces potential procedural variability, as particle size distribution may be influenced by handling and dilution steps. For this reason, the preparation protocol was described in detail to support transparency and reproducibility.

Future research should focus on validating IBMA-based particle characterization in larger and more stratified human milk cohorts for generalizability and to allow for subgroup analyses. In addition, controlled studies examining the effects of specific storage and thawing conditions on particle morphology and aggregation behavior would help clarify the mechanisms underlying the structural changes observed here and their potential nutritional implications.

## 5. Conclusions

In conclusion, IBMA provides a robust and complementary approach to LD for characterizing fat globules in human milk. Unlike bulk measurement methods, IBMA preserves native structures, including clusters, and rapidly analyzes thousands of particles, providing detailed morphological information such as circularity and elongation. This enables the identification and quantification of agglomerates, which represent a significant fraction of the milk fat population. By capturing both particle size and structural heterogeneity in a near-physiological state, IBMA offers valuable insights into the organization of milk fat and its potential impact on digestibility, particularly in premature infants with immature gastrointestinal systems.

## Figures and Tables

**Table 1:** Baseline characteristics of each woman, her infant, and the human milk sampling process.

ID	Age (Years)	Infant Sex	Breastfeeding Duration (Month)	Birth	Time Stored (Day)	Dilution * (IBMA)
1	38	Female	3.3	Full-term	28	No
2	35	Female	18.8	Full-term	17.8	Yes
3	35	Female	3.2	Full-term	19.2	Yes
4	41	Male	12.5	Full-term	19.5	Yes
5	34	Male	5.4	Full-term	24.3	Yes
6	37	Female	5.9	Full-term	11	No
7	35	Female	5.9	Full-term	16.5	No
8	34	Female	12.5	Full-term	11.5	No
9	41	Male	1.3	Preterm	7	No
10	37	Male	14.6	Full-term	7	No
11	19	Male	0.3	Preterm	7	No
12	24	Female	1.5	Preterm	7	No
Mean (SD)	34.2 (6.5)	-	7.1 (6.0)	-	14.7 (7.3)	-

* Sample dilution prior to IBMA application. IBMA—image-based morphometric analysis, SD—Standard deviation.

**Table 2:** Particle number (n) and percentage (%) of total particles, individual particles, and agglomerates determined by image-based morphometric analysis for each woman.

ID	Total	Individual Particles		Agglomerates	
	n	n	%	n	%
1	70,557	60,031	85.08	10,526	14.92
2	107,557	98,324	91.42	9233	8.58
3	74,498	63,371	85.06	10,127	13.59
4	70,582	58,645	83.09	11,937	16.91
5	79,868	72,801	91.15	7067	8.85
6	73,469	60,702	82.62	12,767	17.38
7	88,238	76,803	87.04	11,435	12.96
8	80,960	76,247	94.18	4713	5.82
9	169,546	145,063	85.56	24,483	14.44
10	122,333	104,204	85.18	18,129	14.82
11	132,820	102,742	77.35	30,078	22.65
12	128,048	86,977	67.93	41,071	32.07
Mean (SD)	99,873 (31,978.52)	83,825.83 (25,450.30)	84.75 (6.93)	15,963.83 (10,738.49)	15.25 (6.93)

SD—Standard deviation.

**Table 3:** Number-based circle equivalent diameter metrics and morphological parameters of the 12 human milk samples, measured by image-based morphometric analysis and classified as total particles, individual particles, or agglomerates.

ID	Particle	Circle Equivalent Diameter (µm)				Intensity SD	HS Circularity	Convexity	Elongation	Solidity
		D[1,0]	Pn10	Pn50	Pn90	Pn50	Pn50	Pn50	Pn50	Pn50
1	Total	4.67	2.91	4.25	6.81	27	0.981	0.996	0.040	0.996
	Individual	4.28	2.85	4.00	5.90	33	0.984	0.997	0.032	0.997
	Agglomerates	6.93	4.61	6.55	9.47	36	0.632	0.918	0.386	0.891
2	Total	4.00	2.33	3.54	6.04	27	0.987	0.996	0.023	0.996
	Individual	3.76	2.30	3.42	5.50	27	0.988	0.997	0.021	0.997
	Agglomerates	6.55	3.71	5.88	9.75	31	0.655	0.927	0.384	0.899
3	Total	4.05	2.24	3.34	6.30	27	0.983	0.995	0.037	0.995
	Individual	3.48	2.20	3.15	5.01	27	0.986	0.997	0.031	0.997
	Agglomerates	7.61	3.58	6.41	12.91	35	0.691	0.938	0.352	0.916
4	Total	5.55	3.13	5.05	8.50	28	0.955	0.991	0.052	0.993
	Individual	4.96	3.00	4.71	7.13	28	0.961	0.994	0.050	0.995
	Agglomerates	8.48	5.23	7.91	12.19	31	0.664	0.937	0.385	0.897
5	Total	4.49	2.46	3.72	7.17	30	0.978	0.996	0.050	0.997
	Individual	4.18	2.42	3.58	6.48	30	0.980	0.997	0.046	0.997
	Agglomerates	7.72	3.70	6.36	12.49	35	0.653	0.920	0.336	0.900
6	Total	5.91	3.25	4.14	8.97	33	0.962	0.993	0.055	0.994
	Individual	5.16	3.16	4.72	7.51	33	0.967	0.995	0.046	0.996
	Agglomerates	8.92	5.69	8.20	12.76	43	0.802	0.972	0.507	0.952
7	Total	4.64	2.60	4.00	7.11	32	0.983	0.996	0.040	0.996
	Individual	4.20	2.54	3.77	6.23	31	0.985	0.997	0.035	0.997
	Agglomerates	7.58	4.20	6.50	11.72	36	0.644	0.917	0.343	0.897
8	Total	4.55	2.53	3.95	6.75	27	0.983	0.997	0.043	0.997
	Individual	4.28	2.51	3.85	6.25	27	0.984	0.997	0.041	0.997
	Agglomerates	8.95	4.84	7.23	15.46	32	0.623	0.899	0.320	0.900
9	Total	4.73	2.48	4.25	7.42	29	0.991	0.966	0.016	0.995
	Individual	4.21	2.42	3.95	6.24	28	0.993	0.998	0.013	0.998
	Agglomerates	7.79	5.04	7.49	10.66	35	0.592	0.895	0.397	0.879
10	Total	5.97	3.07	5.44	9.33	38	0.993	0.996	0.011	0.998
	Individual	5.31	2.98	5.01	7.84	37	0.994	0.998	0.008	0.998
	Agglomerates	9.75	6.42	9.40	13.04	43	0.600	0.897	0.396	0.882
11	Total	5.28	3.12	4.39	8.34	35	0.977	0.995	0.031	0.996
	Individual	4.37	3.05	3.97	5.96	34	0.982	0.997	0.023	0.997
	Agglomerates	8.36	5.03	7.21	13.20	39	0.614	0.921	0.517	0.878
12	Total	5.16	3.14	4.54	7.95	35	0.972	0.992	0.042	0.994
	Individual	4.10	3.02	3.90	5.34	34	0.981	0.997	0.025	0.997
	Agglomerates	7.39	5.20	6.96	9.86	37	0.558	0.903	0.429	0.851
Mean (SD)	Total	4.91 (0.66)	2.77 (0.37)	4.21 (0.60)	7.56 (1.06)	30.7 (3.9)	0.978 (0.011)	0.992 (0.009)	0.037 (0.014)	0.996 (0.001)
	Individual	4.36 (0.54)	2.70 (0.34)	4.00 (0.55)	6.28 (0.86)	30.7 (3.5)	0.982 (0.010)	0.997 (0.002)	0.030 (0.013)	0.997 (0.001)
	Agglomerates	8.00 (0.92)	4.77 (0.86)	7.18 (0.98)	11.96 (1.76)	35.6 (3.4)	0.631 (0.036)	0.917 (0.015)	0.385 (0.052)	0.890 (0.016)
*p*	Individual vs. Agglomerates	0.000	0.001	0.000	0.000	0.000	0.000	0.000	0.000	0.000

HS—High sensitivity, SD—Standard deviation.

**Table 4:** Relation between storage duration and birth status on particle size distribution.

Metric	Particle Classification	Storage Duration			
		r		95% CI (LL)	95% CI (UL)
D[1,0]	Total	−0.481		−0.807	−0.053
Pn10	Agglomerates	−0.631		−0.901	−0.318
Pn50	Individual	−0.554		−0.825	−0.183
	Agglomerates	−0.526		−0.812	−0.138
D[4,3]	LD	0.64		0.123	0.918
		**Birth status**			
		**Preterm**	**Full-term**	**95% CI (LL)**	**95% CI (UL)**
D[4,3]	Individual	5.74 (0.87)	7.70 (1.32)	−3.311	−0.725
	Agglomerates	10.99 (2.21)	15.03 (4.38)	−7.618	−0.294
	LD	4.55 (0.67)	8.51 (1.58)	−5.183	−2.699
Pv50	Individual	5.31 (0.57	6.43 (0.97)	−2.048	−0.327
	LD	4.23 (0.65)	5.83 (0.78)	−2.362	−0.693
Pv90	Individual	8.25 (2.15)	13.74 (4.66)	−9.524	−1.749
	Agglomerates	15.30 (3.98)	24.20 (9.35)	−16.589	−1.438
	LD	7.96 (1.20)	16.78 (5.77)	−12.832	−5.004

D[1,0]—number-based circle equivalent mean diameter; Pn10, 50: 10th and 50th percentiles of the circle equivalent number-based particle size distribution. D[4,3]—volume-based equivalent mean diameter; Pv50, 90: 50th, and 90th percentiles of the volume-based particle size distribution. CI—confidence interval, LD—laser diffraction, LL—lower limit, UL—upper limit.

**Table 5:** Mean (SD) and statistical comparison of volume-based equivalent diameter metrics for human milk fat globules as measured by LD and IBMA across three particle classifications.

Metric	Particle Class	IBMA	LD	Pearson Correlation		Paired *t*-Test	CCC	Linear Regression (Proportional Bias *)	
				r	*p* **	*p* **	r_c_	B	95 CI% **
D[4,3]	Total	10.76 (2.76)	7.52 (2.26)	0.476	0.118	0.003	0.246	−0.270	−0.898, 0.530
	Individual	7.21 (1.48)		0.703	0.011	0.505	0.634	0.488	0.159, 1.067
	Agglomerates	14.02 (4.25)		0.573	0.051	0.001	0.159	−0.758	−1.239, −0.142
Pv10	Total	4.46 (0.53)	1.64 (0.52)	0.369	0.238	<0.001	0.022	−0.021	−2.146, 0.499
	Individual	3.75 (0.48)		0.413	0.182	<0.001	0.039	0.109	−1.447, 0.823
	Agglomerates	7.12 (0.91)		0.599	0.040	<0.001	0.017	−0.674	−1.741, −0.111
Pv50	Total	9.17 (1.94)	5.43 (1.02)	0.445	0.147	0.001	0.088	−0.826	−1.488, −0.248
	Individual	6.14 (1.00)		0.849	<0.001	0.003	0.666	0.022	−0.363, 0.361
	Agglomerates	12.74 (3.70)		0.610	0.035	0.006	0.063	−1.307	−1.646, −0.959
Pv90	Total	18.77 (7.06)	14.57 (6.35)	0.818	0.001	0.006	0.671	−0.115	−0.394, 0.383
	Individual	12.37 (4.77)		0.722	0.008	0.122	0.639	0.329	−0.263, 0.863
	Agglomerates	21.98 (9.09)		0.841	0.001	<0.001	0.532	−0.384	−0.603, 0.160

* Differences between the two methods: dependent variable, mean of the two methods: independent variable. ** 3000 bias-corrected and accelerated bootstrap samples were utilized; procedure offered within the SPSS package. D[4,3]—volume-based equivalent mean diameter; Pv10, 50, 90: 10th, 50th, and 90th percentiles of the equivalent volume-based particle size distribution. B—slope of the linear regression for proportional bias; CCC—Lin’s concordance correlation coefficient; CI—confidence interval; IBMA—image-based morphometric analysis; LD—laser diffraction, SD—standard deviation.

## Data Availability

The original contributions presented in the study are included in the article/Supplementary Materials. Further inquiries can be directed to the corresponding author.

## Supplementary Materials

The following supporting information can be downloaded at: https://www.mdpi.com/article/10.3390/foods15071205/s1. Figure S1. Box plot comparison of particle size distribution between LD and IBMA for individual particles. Figure S2. Box plot comparison of particle size distribution between LD and IBMA for agglomerates. Figure S3. Box plot comparison of particle size distribution between LD and IBMA for total particles.

## Abbreviations

The following abbreviations are used in this manuscript:

CE	Circle equivalent
HS	High sensitivity
IBMA	Image-based morphometric analysis
LD	Laser diffraction
MFG	Milk fat globule
SD	Standard deviation
SOP	Standard operating procedure

## References

[1] Brink L.R., Lönnerdal B. (2020). Milk Fat Globule Membrane: The Role of Its Various Components in Infant Health and Development. J. Nutr. Biochem..

[2] Lee H., Padhi E., Hasegawa Y., Larke J., Parenti M., Wang A., Hernell O., Lönnerdal B., Slupsky C. (2018). Compositional Dynamics of the Milk Fat Globule and Its Role in Infant Development. Front. Pediatr..

[3] Donda K., Maheshwari A. (2022). Human Milk Lipids Induce Important Metabolic and Epigenetic Changes in Neonates. Clin. Perinatol..

[4] Thum C., Wall C., Day L., Szeto I.M.Y., Li F., Yan Y., Barnett M.P.G. (2022). Changes in Human Milk Fat Globule Composition Throughout Lactation: A Review. Front. Nutr..

[5] Michalski M.C., Briard V., Michel F., Tasson F., Poulain P. (2005). Size Distribution of Fat Globules in Human Colostrum, Breast Milk, and Infant Formula. J. Dairy Sci..

[6] Thum C., Roy N.C., Everett D.W., McNabb W.C. (2023). Variation in Milk Fat Globule Size and Composition: A Source of Bioactives for Human Health. Crit. Rev. Food Sci. Nutr..

[7] Martínez-Sánchez V., Fontecha J., Pérez-Gálvez A. (2024). Milk Fat Globule Membrane: Production, Digestion, and Health Benefits Evaluated through in Vitro Models. PharmaNutrition.

[8] Lopez C., Ménard O. (2011). Human Milk Fat Globules: Polar Lipid Composition and in Situ Structural Investigations Revealing the Heterogeneous Distribution of Proteins and the Lateral Segregation of Sphingomyelin in the Biological Membrane. Colloids Surf. B Biointerfaces.

[9] Garcia C., Antona C., Robert B., Lopez C., Armand M. (2014). The Size and Interfacial Composition of Milk Fat Globules Are Key Factors Controlling Triglycerides Bioavailability in Simulated Human Gastro-Duodenal Digestion. Food Hydrocoll..

[10] Ramiro-Cortijo D., Singh P., Liu Y., Medina-Morales E., Yakah W., Freedman S.D., Martin C.R. (2020). Breast Milk Lipids and Fatty Acids in Regulating Neonatal Intestinal Development and Protecting against Intestinal Injury. Nutrients.

[11] Burge K., Vieira F., Eckert J., Chaaban H. (2021). Lipid Composition, Digestion, and Absorption Differences among Neonatal Feeding Strategies: Potential Implications for Intestinal Inflammation in Preterm Infants. Nutrients.

[12] Dassoff E., Shireen A., Wright A. (2025). Lipid Emulsion Structure, Digestion Behavior, Physiology, and Health: A Scoping Review and Future Directions. Crit. Rev. Food Sci. Nutr..

[13] Bihola A., Suvera P., Jana A.H., Pratiksha, Patwadi P., Chaudhary M.B., Adil S. (2026). Microstructural Characterization of Dairy Products: Structure–Function Relationships, Processing Effects, and Industrial Significance. Discov. Food.

[14] Verveld W., De Wolf J.R., Legtenberg C.G., Knop T., Bosschaart N. (2024). Human Milk Fat Globule Size Distributions: Comparison between Laser Diffraction and 3D Confocal Laser Scanning Microscopy. Food Res. Int..

[15] Ozturk G., Paviani B., Rai R., Robinson R.C., Durham S.D., Baller M.I., Wang A., Nitin N., Barile D. (2024). Investigating Milk Fat Globule Structure, Size, and Functionality after Thermal Processing and Homogenization of Human Milk. Foods.

[16] Jiang W., Zhang X., Cheng J., Song J., Jin Q., Wei W., Dong M., Wang X. (2020). Variation of Fat Globule Size and Fatty Acids in Human Milk in the First 30 Days of Lactation. Int. Dairy J..

[17] Pan Y., Liu L., Tian S., Li X., Hussain M., Li C., Zhang L., Zhang Q., Leng Y., Jiang S. (2022). Comparative Analysis of Interfacial Composition and Structure of Fat Globules in Human Milk and Infant Formulas. Food Hydrocoll..

[18] Zou X.-Q., Guo Z., Huang J.-H., Jin Q.-Z., Cheong L.-Z., Wang X.-G., Xu X.-B. (2012). Human Milk Fat Globules from Different Stages of Lactation: A Lipid Composition Analysis and Microstructure Characterization. J. Agric. Food Chem..

[19] Wei W., Cheng J., Yang J., Chen C., Jin Q., Song J., Wang X. (2021). Phospholipid Composition and Fat Globule Structure Change during Low Temperature Storage of Human Milk. LWT.

[20] Nebbia S., Giribaldi M., Cavallarin L., Bertino E., Coscia A., Briard-Bion V., Ossemond J., Henry G., Ménard O., Dupont D. (2020). Differential Impact of Holder and High Temperature Short Time Pasteurization on the Dynamic in Vitro Digestion of Human Milk in a Preterm Newborn Model. Food Chem..

[21] Malvern (2015). A Basic Guide to Particle Characterization; Malvern Instruments Limited. https://www.malvernpanalytical.com/en/learn/knowledge-center/whitepapers/wp120620basicguidepartchar.

[22] Bittelli M., Andrenelli M.C., Simonetti G., Pellegrini S., Artioli G., Piccoli I., Morari F. (2019). Shall We Abandon Sedimentation Methods for Particle Size Analysis in Soils?. Soil Tillage Res..

[23] Malvern (2007). Morphologi G3 Automated Microscope System. https://photos.labwrench.com/equipmentManuals/2665-520.pdf.

[24] Malvern Panalytical (2014). Identification of Agglomerates Using Automated Image Analysis.

[25] Huppertz T., Uniacke-Lowe T., Kelly A.L., McSweeney P.L.H., Fox P.F., O’Mahony J.A. (2020). Physical Chemistry of Milk Fat Globules. Advanced Dairy Chemistry, Volume 2: Lipids.

[26] Kupikowska-Stobba B., Niu H., Klojdová I., Agregán R., Lorenzo J.M., Kasprzak M. (2025). Controlled Lipid Digestion in the Development of Functional and Personalized Foods for a Tailored Delivery of Dietary Fats. Food Chem..

[27] Liu D., Zhang C., Chen Z., Zhang X., Han X., Chen L., Hu J., Zhou P. (2023). Effect of Cold Storage on Human Milk Fat Globule Membrane: Microstructure and Proteomic Analysis. Food Biosci..

[28] Walstra P., Walstra P., Wouters J.T.M., Geurts T.J. (2005). Dairy Science and Technology.

[29] Ballard O., Morrow A.L. (2013). Human Milk Composition: Nutrients and Bioactive Factors. Pediatr. Clin. N. Am..

[30] Ingvordsen Lindahl I.E., Artegoitia V.M., Downey E., O’Mahony J.A., O’Shea C.A., Ryan C.A., Kelly A.L., Bertram H.C., Sundekilde U.K. (2019). Quantification of Human Milk Phospholipids: The Effect of Gestational and Lactational Age on Phospholipid Composition. Nutrients.

[31] HORIBA Particle Size Result Interpretation: Number vs. Volume Distributions. HORIBA Scientific.

[32] Looney M.A. (2018). Assessment of Interrater and Intermethod Agreement in the Kinesiology Literature. Meas. Phys. Educ. Exerc. Sci..

[33] Wadoux A.M.J.-C., Minasny B. (2024). Some Limitations of the Concordance Correlation Coefficient to Characterise Model Accuracy. Ecol. Inform..

[34] Bourlieu C., Michalski M.-C. (2015). Structure–Function Relationship of the Milk Fat Globule. Curr. Opin. Clin. Nutr. Metab. Care.

