# Supplementary material for: Image-Based Morphometric Analysis of Human Milk Fat Globules Versus Laser Diffraction
Source: Foods. 2026 Apr 2;15(7):1205. doi: 10.3390/foods15071205 (PMC13073344; doi:10.3390/foods15071205)
Supplement: Supplementary file 1 [file foods-15-01205-s001.zip › foods-4208910-supplementary.pdf]

foods

MDPI

Article

Image-Based Morphometric Analysis of Human Milk Fat Globules Versus Laser Diffraction

Diana Escuder-Vieco [1,2,*,†], Kristin Keller [1,2,†], Noelia Ureta-Velasco [3], Clara Alonso-Díaz [2,3], María López Cerdán [3], Carmen Rosa Pallás-Alonso [2,3] and Nadia Raquel García-Lara [1,2,3]

[1] Aladina-MGU Regional Human Milk Bank, 12 de Octubre University Hospital, imas12, 28041 Madrid, Spain; biol.kristin.keller@gmail.com (K.K.); nadiaraquelg.nrgl@gmail.com (N.R.G.-L.)
[2] Spanish Network in Maternal, Neonatal, Child and Developmental Health Research (RICORS-SAMID, RD24/0013/0008), Instituto de Salud Carlos III, 28029 Madrid, Spain; claraalonsodiaz@gmail.com (C.A.-D.); kpallas.hdoc@gmail.com (C.R.P.-A.)
[3] Department of Neonatology, 12 de Octubre University Hospital, 28041 Madrid, Spain; noelia.ureta@gmail.com (N.U.-V.); mlcerdan@salud.madrid.org (M.L.C.)
* Correspondence: diana.e.vieco@gmail.com
† These authors contributed equally to this work.

1. Supplementary Materials

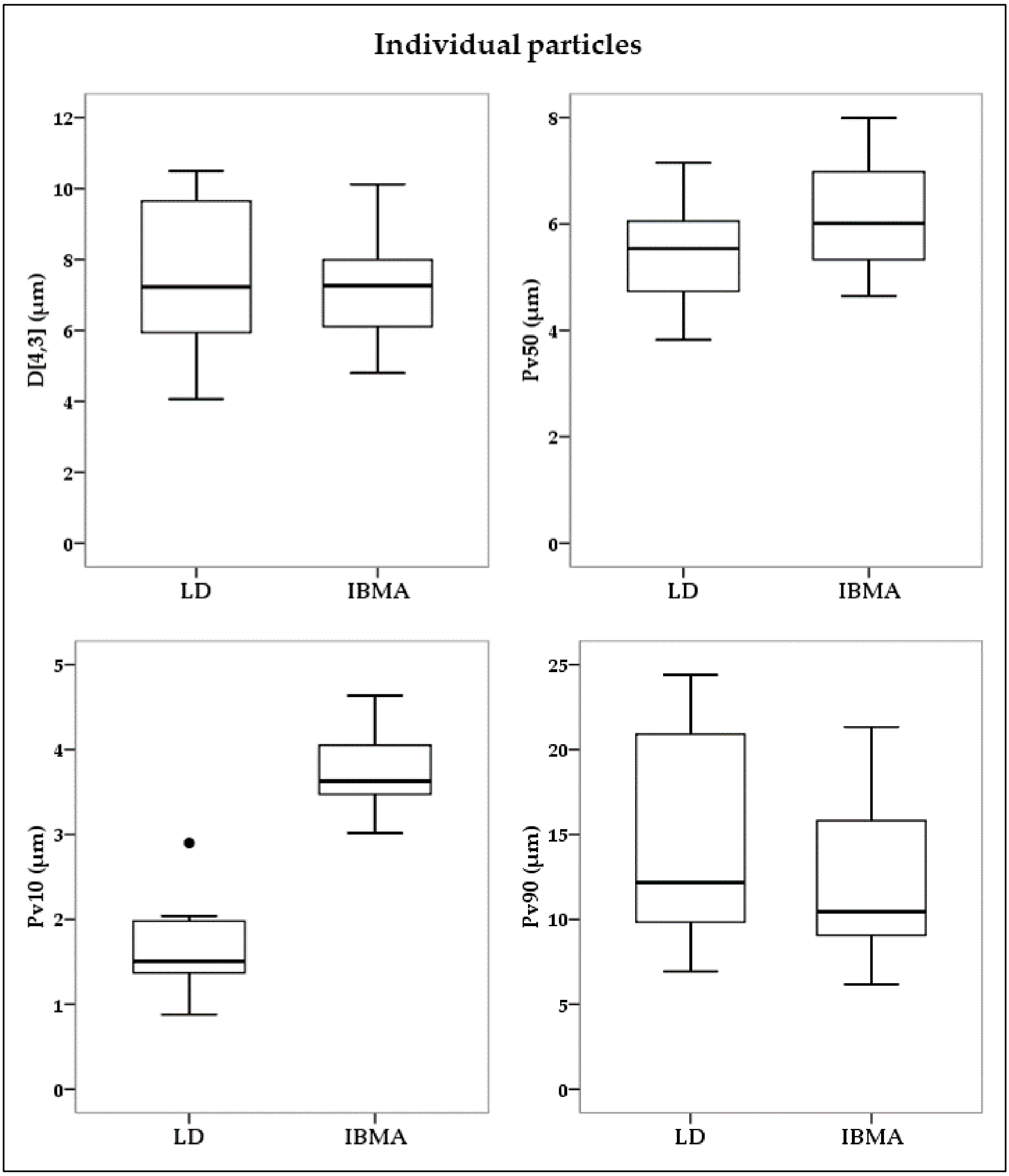

Figure S1. Box plot comparison of particle size distribution between LD and IBMA for individual particles. Data are presented as boxplots of the volume-weighted mean diameter (D[4,3]) and volume percentiles (Pv10, Pv50, Pv90) for LD and IBMA methods. Within each plot, the central horizontal line represents the median, the box edges denote the interquartile range, and the whiskers indicate the minimum and maximum values. LD – laser diffraction, IBMA – image-based morphological analysis.

https://doi.org/10.3390/foods15071205

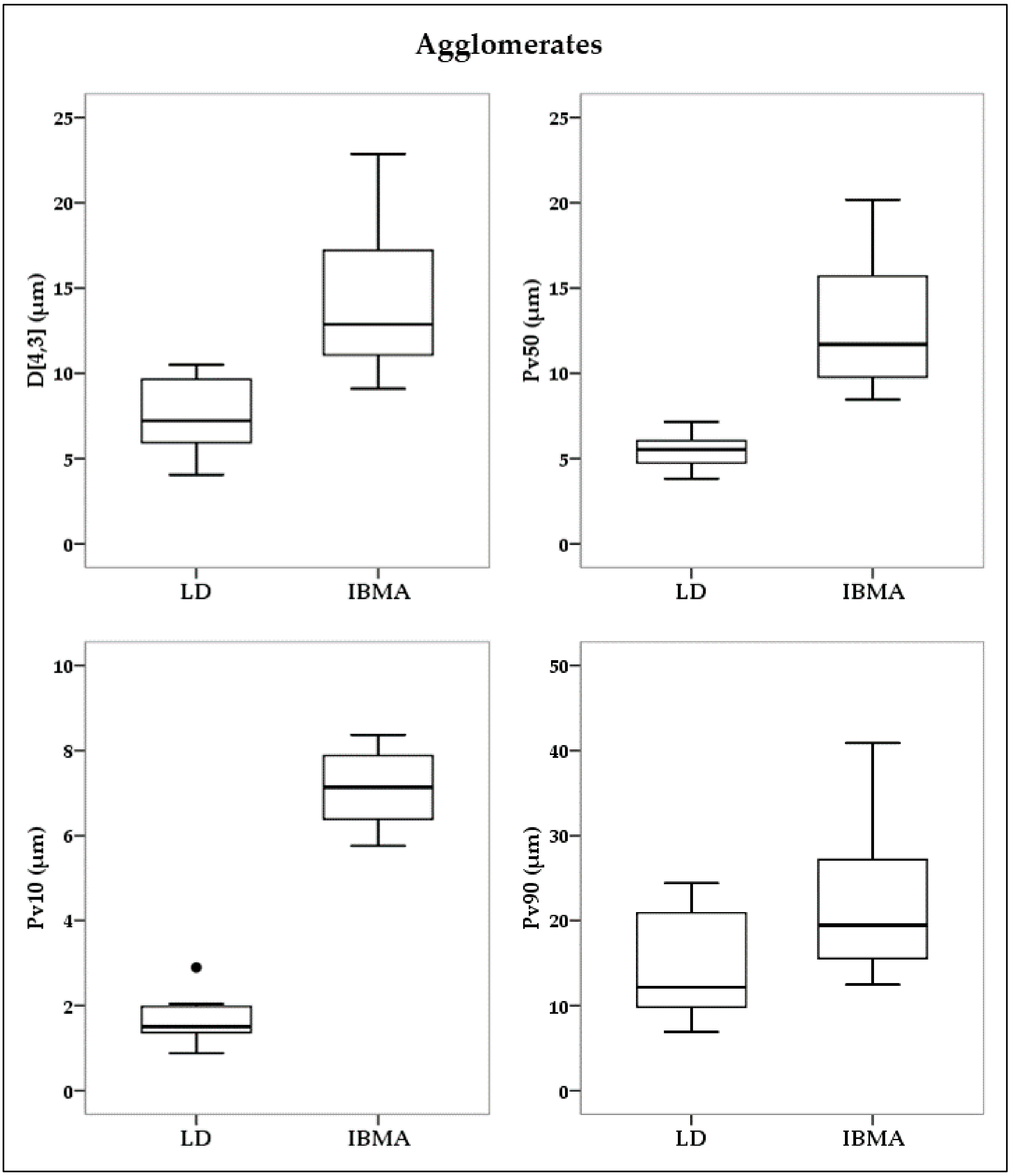

Figure S2. Box plot comparison of particle size distribution between LD and IBMA for agglomerates. Data are presented as box-plots of the volume-weighted mean diameter (D[4,3]) and volume percentiles (Pv10, Pv50, Pv90) for LD and IBMA methods. Within each plot, the central horizontal line represents the median, the box edges denote the interquartile range, and the whiskers indicate the minimum and maximum values. LD – laser diffraction, IBMA – image-based morphological analysis.

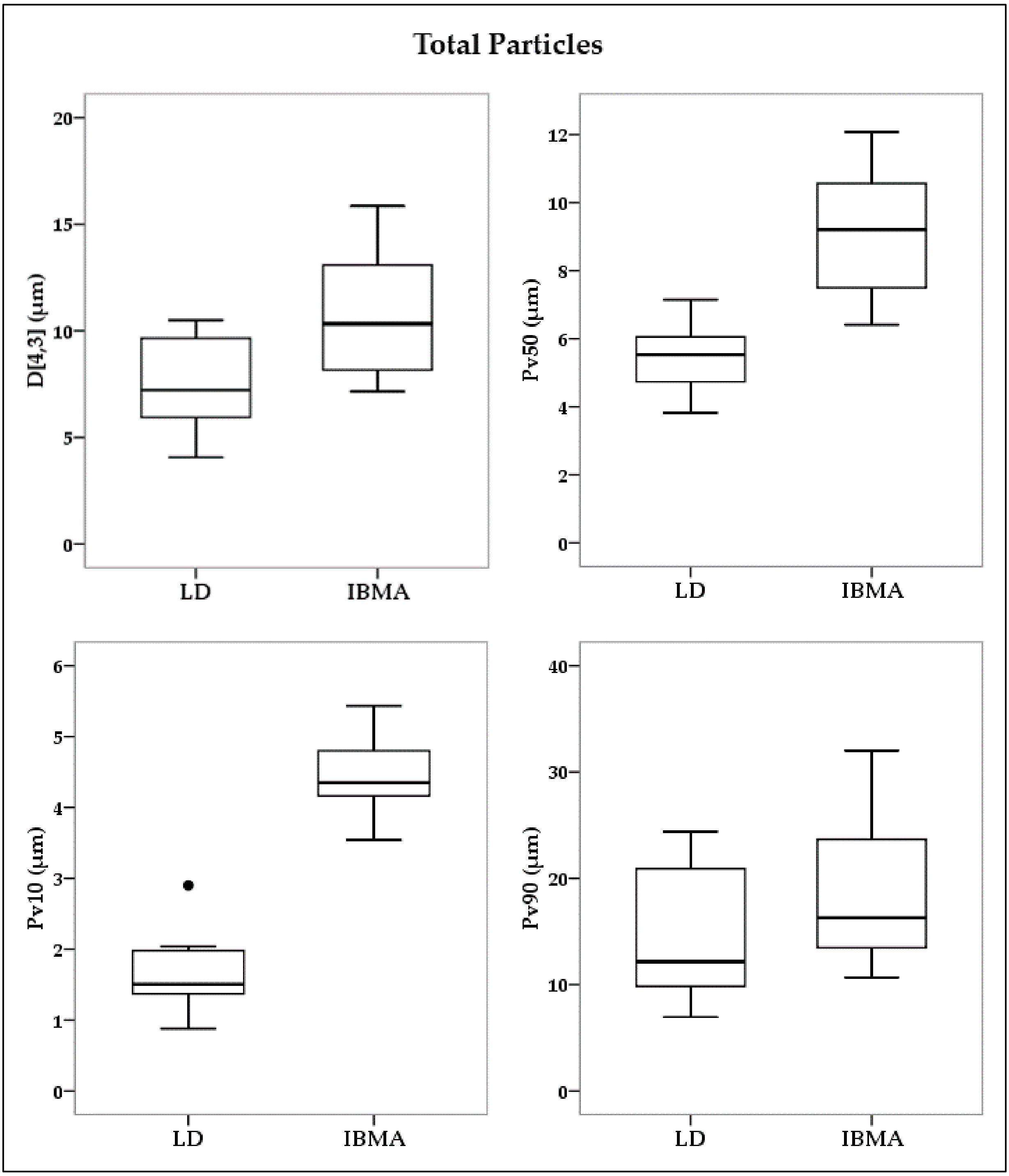

Figure S3. Box plot comparison of particle size distribution between LD and IBMA for total particles. Data are presented as boxplots of the volume-weighted mean diameter (D[4,3]) and volume percentiles (Pv10, Pv50, Pv90) for LD and IBMA methods. Within each plot, the central horizontal line represents the median, the box edges denote the interquartile range, and the whiskers indicate the minimum and maximum values. LD – laser diffraction, IBMA – image-based morphological analysis.

https://doi.org/10.3390/foods15071205
